# Clinical Results after High-Dose Intensity-Modulated Radiotherapy for High-Risk Prostate Cancer

**DOI:** 10.1155/2012/368528

**Published:** 2011-11-30

**Authors:** Valérie Fonteyne, Nicolaas Lumen, Geert Villeirs, Piet Ost, Gert De Meerleer

**Affiliations:** ^1^Department of Radiotherapy, Ghent University Hospital, 9000 Ghent, Belgium; ^2^Department of Urology, Ghent University Hospital, 9000 Ghent, Belgium; ^3^Department of Radiology, Ghent University Hospital, 9000 Ghent, Belgium

## Abstract

*Purpose*. Patients with high-risk prostate cancer (PC) can be treated with high-dose intensity-modulated radiotherapy (IMRT) and long-term androgen deprivation (AD). In this paper we report on (i) late toxicity and (ii) biochemical (bRFS) and clinical relapse-free survival (cRFS) of this combined treatment. *Methods*. 126 patients with high-risk PC (T3-4 or PSA >20 ng/mL or Gleason 8–10) and ≥24 months of followup were treated with high-dose IMRT and AD. Late toxicity was recorded. Biochemical relapse was defined as PSA nadir +2 ng/mL. Clinical relapse was defined as local failure or metastases. *Results*. The incidence of late grade 3 gastrointestinal and genitourinary toxicity was 2 and 6%, respectively. Five-year bRFS and cRFS were 73% and 86% respectively. AD was a significant predictor of bRFS (*P* = 0.001) and cRFS (*P* = 0.01). *Conclusion*. High-dose IMRT and AD for high-risk PC offers excellent biochemical and clinical control with low toxicity.

## 1. Introduction

The of PSA screening has resulted in an increased detection rate of prostate cancer (PC) with stage migration towards lower-stage prostate cancer (PC). Nevertheless, still 12% of the patients with PC will have locally advanced (T3-4 N0 M0 or Tx N1 M0) or metastatic disease at diagnosis [1]. More aggressive therapies are indicated for these patients as they are at increased risk of PC death [2]. External beam radiotherapy (EBRT) is one of the standard treatment options of choice for those patients. However, when conventional low-dose (<72 Gy) EBRT is applied in patients with clinical stage T3-T4 PC, local recurrence rates mount to 30% at 10 years [3]. Improvement of local control is important as local failure is directly correlated with distant metastasis [3, 4] and survival [5]. Extensive evidence exists that high-dose radiotherapy (dose ≥ 74 Gy) is superior to conventional dose radiotherapy (dose 64–70 Gy) [6–8]. For high-risk patients, an increase in 5-year biochemical relapse-free survival (bRFS) of 19% has been reported when increasing the dose from 70 Gy to 80 Gy [9]. Zelefsky et al. demonstrated that the rate of positive biopsies after EBRT dropped with 30% when the dose was increased from <70.2 Gy to >81 Gy [10]. With modern radiotherapy techniques, such as intensity-modulated radiotherapy (IMRT), dose escalation can safely be performed [11]. Randomized trials support the combined use of EBRT and androgen deprivation (AD) with superior disease-specific and overall survival outcomes in patients with locally advanced-stage or high-risk disease [12, 13].

Based on the above-mentioned data, patients with high-risk PC are treated at our institute with high-dose IMRT and 24–36 months of AD. In this paper, we report on

- late gastrointestinal (GI) and genitourinary (GU) toxicity,
- biochemical control,
- clinical control

of this combined treatment modality.

## 2. Material and Methods

### 2.1. Patients

Between December 1998 and March 2011, 604 patients were treated with IMRT as primary therapy for prostate cancer at Ghent University Hospital (GUH). Over time 3 different dose levels were initiated as has been previously reported [14, 15]. Between 1996 and 2001, 2 prescriptions were launched: 74 Gy (74R72) and 76 Gy (76R74) as median dose to the planning target volume (PTV) of the prostate + seminal vesicles with a hard constraint on maximal rectal dose of 72 and 74 Gy, respectively. In 2002, a third dose escalation level was initiated, in which we treated the PTV to 78 Gy while keeping the maximal rectal dose at 76 Gy (78R76).

High-risk PC was defined as PC with one of the following characteristics: clinical T3-4 or PSA >20 ng/mL or Gleason 8–10. Patients with pN1 or cN1 disease were treated within another study protocol [16] and consequently not included in this study. In total 43% of the patients fulfilled these criteria. Only patients with high-risk PC and a minimal followup of 24 months were considered for this report resulting in a study population of 126 patients: 21 patients in the 74R72 group, 19 patients in the 76R74 group, and 86 patients in the 78R76 group.

T stage was determined by digital rectal examination supplemented with magnetic resonance (MR) imaging data. The 2002 American Joint Commission on Cancer staging was used [17]. Lymph node staging was done by CT scan in all cases and in 54 cases by pelvic lymphadenectomy. All patients underwent bone scintigraphy. Except for 10 patients, who refused AD, a luteinizing-hormone releasing hormone (LHRH) analogue was initiated for a period of 24–36 months.

A fixed questionnaire was used to register the medical history and pretreatment GU and GI symptoms of each patient.

### 2.2. Treatment Planning

Details on pretreatment imaging, delineation of clinical target volume (CTV) and organs at risk, expansion of CTV to PTV, treatment planning, criteria for plan acceptance, leaf position optimization, patient preparation, and treatment delivery can be found in our previous work [14]. In brief, the CTV consisted of the prostate and seminal vesicles. None of the patients received elective lymph node irradiation. The PTV was created using a 3-dimensional, isotropic expansion of the CTV of 7 mm. The rectal wall (excluding air and faeces), sigmoid colon, bladder, small bowel, and femoral heads were delineated as organs at risk. Patients were treated with empty rectum and comfortably filled bladder.

The dose was prescribed as the median dose to the PTV. Treatment was delivered using 18 MV photons of an Elekta linear accelerator (Crawley, UK) equipped with a multileaf collimator (MLC) and able to deliver IMRT in a step-and-shoot mode. Since 2009, patients were also treated on a Clinac ix (Varian Medical Systems, Palo Alto, Calif, USA). Until 2009, 3 beams with gantry angles 0°, 116°, and 244° were used [8]. Thereafter, planning was performed with 7 beams (gantry angles: 0°, 52°, 103°, 154°, 206°, 257°, and 308°) or with single arc therapy (1 full arc counter clockwise) [18]. First, a fixed couch height and portal imaging procedure (Elekta electronic portal imaging device) was used to correct patient positioning. Thereafter, an ultrasound-based (SonArray, Zmed, Ashland, USA), prostate positioning was added to correct for prostate positioning. Since 2009, positioning is performed by daily kilovoltage cone beam CT.

### 2.3. Followup

Patients were seen every 3 months for the first year, biannually until 5 years, and yearly thereafter. A fixed toxicity questionnaire was fulfilled at each visit.

### 2.4. End Points

*Late toxicity* was defined as any increase of any GI or GU toxicity lasting more than 3 months after cessation of IMRT or occurring for the first time later than 3 months after the end of IMRT. The grade of late GI (Table 1(a)) and GU toxicity (Table 1(b)) was scored according to an in-house developed scoring system based on the RTOG, SOMA/LENT, and CTC toxicity scorings system [19, 20]. For each symptom, the maximal toxicity score was registered.

*Biochemical relapse* was defined according to the Phoenix consensus definition, that is, PSA nadir +2 ng/mL [21].

*Clinical relapse* was defined as local failure (determined on prostate biopsies) or metastases (both lymph node and haematogenous metastasis) detected on imaging (18F-fluorodeoxyglucose positron-emitting tomography/computed tomography and bone scan) performed at the time of biochemical relapse.

Kaplan-Meier statistics were used to report on 5-year bRFS and clinical relapse-free survival (cRFS). Univariate analysis (log-rank test) was used to examine the predictive value of the dose prescription group, Gleason score group (Gleason 6 versus 7 versus 8–10), cT (T1-T2-T3-T4), PSA (PSA < 10 ng/mL versus PSA: 10–20 ng/mL versus PSA ≥ 20), staging by lymphadenectomy (pN0 versus pNx), and use of AD. Multivariate analysis was performed using Cox regression analysis.

Using *Chi-square statistics*, the baseline patient-related risk factors were compared for the different prescription groups. A *P* value of < 0.05 was considered statistically significant.

Statistical analysis was performed with SPSS version 15.0 software (Chicago, ILL, USA).

## 3. Results

Patient characteristics and planning parameters are shown in Tables 2 and 3, respectively. Significantly fewer patients received AD in prescription group 74R72 (*P* = 0.002). Except for follow-up time, all other parameters were equally balanced between the different prescription groups. Median followup was 48 months.

### 3.1. Late Toxicity

Late toxicity was mild. No patient developed grade 4 GI or GU toxicity. The incidence of grade 1–3 late GI and GU toxicity is presented in Table 4. The crude incidence of late grade 3 GI and GU toxicity was 2 and 6%, respectively. Dose escalation did not result in increased GI or GU toxicity.

### 3.2. Biochemical Relapse

**Figure 1:**
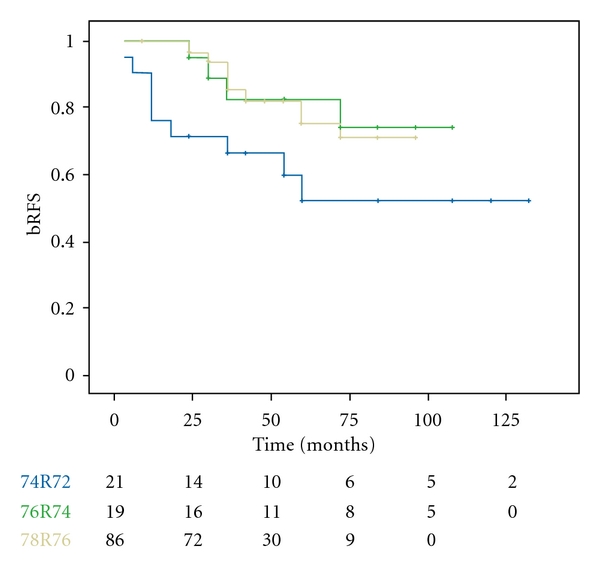
Biochemical relapse-free survival according to prescription group.

Twenty-eight patients experienced biochemical relapse resulting in a 5-year bRFS of 73% for the whole group. T stage, addition of lymphadenectomy, pretreatment PSA, and Gleason score were not significantly correlated with bRFS. Although not significant, there was a strong trend towards better 5-year bRFS rates with higher radiotherapy doses (52%, 83%, and 76% for 74R72, 76R74, and 78R76, resp., *P* = 0.051) (Figure 1).

**Figure 2:**
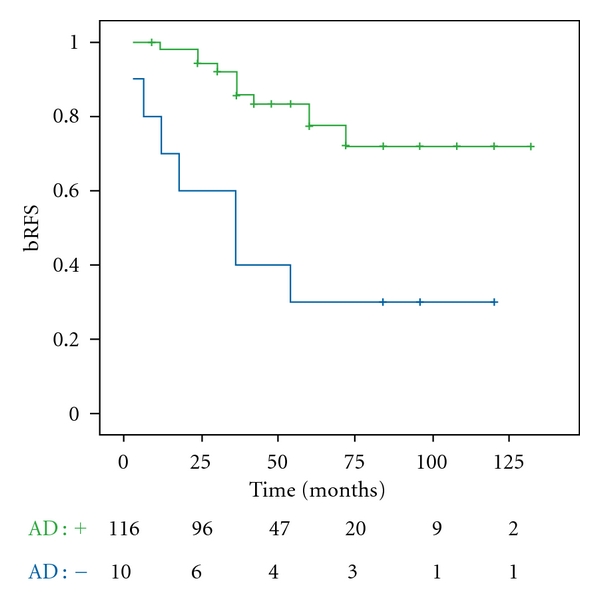
Biochemical relapse-free survival for patients treated with high-dose IMRT with or without androgen deprivation.

 The association of AD was significantly correlated with 5-year bRFS (77% versus 30%; *P* < 0.001) (Figure 2).

In multivariate analysis AD remained a significant predictor of bRFS (*P* = 0.001).

### 3.3. Clinical Relapse

Fourteen patients had a clinical relapse. Clinical relapses occurred in the lymph nodes (*N* = 4), bone (*n* = 9), or prostate (*n* = 2). One patient had both lymph node and bone metastases at time of clinical relapse. The 5-year cRFS was 86%. T stage, addition of lymphadenectomy, pretreatment PSA, and Gleason did not significantly influence cRFS. There was a significant correlation between 5-year cRFS and dose (67%, 83%, and 90% for 74R72, 76R74, and 78R76, resp.; *P* = 0.04) as well as between 5-year cRFS and AD (91% versus 30%; *P* < 0.001).

The only significant predictor of cRFS in multivariate analysis was AD (*P* = 0.01).

## 4. Discussion

Multiple treatment options are available for patients with high-risk PC such as surgery, high-dose EBRT, and AD.

AD has been the primary treatment for patients with high-risk PC for many years. Although the response rate is high, AD alone is not a curative therapy and has important side effects. A recently published randomized trial confirmed that the addition of EBRT to AD resulted in a significant improvement in overall survival when compared to AD [22].

Merglen et al. claimed that surgery, as a single treatment, offers the best 10-year survival rates for T1–T3 PC patients when compared to EBRT without AD, particularly for younger patients and patients with poorly differentiated tumours [23]. However this study has major shortcomings such as the lack of information on radiation dose and an imbalance between the surgery and radiotherapy group concerning Gleason score and PSA [23]. Moreover, 10-year overall survival and PC-specific survival was better for patients treated with EBRT and AD versus prostatectomy alone (80% versus 69% and 87% versus 83% for overall survival and PC-specific survival, resp.).

 Akakura et al. randomized patients between radical prostatectomy and low-dose EBRT (60–70 Gy) both combined with AD. The 10-year overall survival rates were better for the surgery group, although not statistically significant [24]. Recently the long-term outcome of patients with high-risk PC was reported comparing survival after RP and EBRT ± AD. The authors concluded that RP and EBRT + AD provided similar long-term cancer control for patients with high-risk PC [25]. In a retrospective matched case analysis, RP, brachytherapy, and multimodality radiotherapy (i.e., EBRT with brachytherapy boost and AD) were compared. Significantly improved bRFS at 4 years was reported with multimodality radiotherapy (multimodality radiotherapy: 72%, brachytherapy: 25%, and RP: 53%, *P* < 0.001) [26]. In the absence of randomized trials and based on published data surgery, whether or not combined with adjuvant radiotherapy and high-dose radiotherapy combined with AD should be considered as equally effective in this patient group.

In the published series, clinical or biochemical relapse is still observed in more than a quarter of the patients 5 years after treatment. Extensive evidence exists that high-dose radiotherapy (dose ≥ 74 Gy) is superior to conventional dose radiotherapy (dose 64–70 Gy). Zelefsky et al. reported long-term results after high-dose radiotherapy for T3 PC. For patients treated with high doses (81 Gy) combined with AD, 5- and 10-year PSA relapse-free survival was 77% and 52% for T3a stage and 53% and 49% for T3b stage PC. Dose was an important predictor of improved biochemical control. With higher doses (≥ 81 Gy), 5- and 10-year local progression-free survival of 96% and 88% is reported [27].

Our data confirm these encouraging figures with 5-year bRFS and cRFS rates of 73% and 86%, respectively. In contrast with the study of Zelefsky et al., we were not able to detect a significant dose-response relationship. Although there was a significant relation between prescription dose and cRFS and a strong trend towards better bRFS with higher doses, this was no longer present in multivariate analysis. These data must be interpreted with caution due to the small number of patients in the lowest prescription group as well as the imbalance between the patients receiving AD in the different prescription groups. Significantly fewer patients in prescription group 74R72 received AD. The role of concomitant AD was unequivocally confirmed in our study with a significant impact on bRFS and cRFS in uni- and multivariate analysis.

Dose escalation to the prostate is only defendable if both radiotherapy-induced GI and GU toxicities remain acceptable. There is level 1 evidence that toxicity increases with dose when conventional or conformal radiation technologies are used [11]. The implementation of new radiotherapy technologies has resulted in low GI toxicity rates. Even with dose escalation to the prostate late grade ≥3 GI toxicity is rare with modern radiotherapy techniques with incidence rates of <1% [28] to 2% [29] and 5% [30]. On the contrary, GU toxicity is more frequent with incidence rates of late grade ≥ 3 GU toxicity of 13% [30]. The reported toxicity rates in our study (grade 3 GI: 2% and grade 3 GU: 6%) are comparable with published data and confirm that high-dose IMRT combined with AD can safely be delivered. Dose escalation did not result in higher toxicity rates in our study probably as a result of the implementation of a direct aperture and weight optimization (SOWAT) in the higher prescription groups. In a planning study, the use of SOWAT resulted in a reduction of the rectal complication probability by lowering the physical dose to rectal volumes without compromising the dose to the prostate. The present paper confirms that SOWAT is clinically relevant and makes further dose escalation possible without increasing rectal or urinary toxicity.

The role of prophylactic pelvic irradiation for patients with high-risk PC is still under debate. Two large randomised trials were published with opposite results. The Radiation Therapy Oncology Group (RTOG) 9413 trial favours pelvic radiotherapy [31]. A significant 7% improvement in the 4-year progression-free survival (PFS) rate was reported when patients were treated with a combination of neoadjuvant + concurrent AD and pelvic EBRT compared with prostate-alone EBRT for patients with intermediate and high-risk PC. However, there was no significant benefit in overall or distant metastases-free survival. Importantly, an increase in late grade 2 and 3 toxicities was noted [31].

The GETUG randomised trial on the contrary failed to show differences in PFS [32].

There are 2 important shortcomings of these “older” trials that might have influenced the results: at first, the radiotherapy dose to the prostate was low (70 Gy). Secondly there might have been an insufficient coverage of the pelvic lymph nodes regions at risk. The role of dose was evaluated in the GETUG trial in which they failed to show a significant difference in the groups receiving < or ≥ 70 Gy at the level of the prostate, which is, after all, still a low dose [32].

A large retrospective study with high-dose brachytherapy also failed to demonstrate a benefit for pelvic irradiation, suggesting that dose escalation to the prostate rather than pelvic radiotherapy is beneficial [33].

A new phase III trial (RTOG 0924) will soon be opened for enrolment further addressing the issue on prophylactic pelvic irradiation. The RTOG 0924 is a phase III trial for intermediate and favourable high-risk PC patients randomizing between androgen deprivation and high-dose radiotherapy with or without whole pelvic radiotherapy. PIVOTAL is another multicentre study for patients with locally advanced PC randomising between high-dose IMRT to the prostate ± pelvic lymph nodes. The endpoints of the study are toxicity, quality of life, and disease outcome. Patient recruitment is now ongoing.

In the absence of the results of these “modern” phase III trials, the implementation of pelvic irradiation is not current standard and left at the discretion of the radiotherapists. In our study only 4 patients had a clinical relapse in the lymph nodes making the omission of prophylactic pelvic radiotherapy defendable certainly when taking into account the increased risk of GI toxicity as a result of irradiation of larger volumes of small bowel, even with modern radiotherapy techniques.

Some recent data suggest that the patient's outcome is positively influenced by staging lymphadenectomy. However, the exact impact of an extended lymphadenectomy on patient outcomes has not yet been clearly determined. Recently, Masterson et al. reported that a higher number of nodes removed correlated significantly with bRFS in men without nodal involvement [34] probably as a result of elimination of micrometastases that are not detected by routine histological examination. Joslyn and Konety [35] published similar results. Patients included in this study were treated since 1996. At that time staging lymphadenectomies were not routinely performed. Consequently only few patients in our study received a staging pelvic lymphadenectomy. Moreover, there is an important lack of information on extent of lymphadenectomy and number of lymph nodes removed. In our study 3 of the 4 patients presenting with lymph node relapse did not have previous lymphadenectomy.

## 5. Conclusion

High-dose IMRT and AD for high-risk PC offers excellent biochemical and clinical control with low toxicity.

## Figures and Tables

**Table:** (a)

GI	Grade 1	Grade 2	Grade 3	Grade 4
Abdominal cramps	Present, no therapy	Peroral therapy, for example, Spasmolytic	IV therapy	Surgery
Diarrhea	Present, no therapy	Peroral therapy, for example, loperamide	IV therapy	Surgery
Frequency	Present, no therapy	Peroral therapy, for example, loperamide	IV therapy	—
Mucus loss	Present, no therapy	Need hygienic pads	Continuous, invasive therapy	Surgery
Red blood loss	No therapy, frequency < 3x/week	Frequency ≥ 3x/week	Invasive therapy needed, for example, laser coagulation	Transfusion need, surgery
Urgency	Present, no therapy	Peroral therapy	IV therapy	—
Incontinence	Present, no therapy	Need hygienic pads (≤2/day)	Need hygienic pads (>2/day)	Surgery
Anal pain	Present, no therapy	Local anesthetic for example, Xylogel	Narcotic analgetica	Surgery

**Table:** (b)

GU	Grade 1	Grade 2	Grade 3	Grade 4
Nocturia	Twice pretherapy, 2-3 times	4–6 times (<1x hour)	>6 times (more frequently than hourly)	—
Frequency	Once/2 h, twice pretherapy	Once/1 h	Once/0.5 h (more frequent than hourly)	—
Hematuria	Microscopic	Intermittent/moderate	Frequent, gross hematuria/minor surgery needed (coagulation)	Hemorrhagic cystitis requiring transfusion/ ulceration/ necrosis
Dysuria	Slight, no medication	Moderate, requiring local anesthetic (including bladder spasm)	Dysuria, regular and frequent narcotics needed (including bladder spasm and pelvis pain)/severe/ stenosis/TUR or dilatation	Bladder obstruction not secondary to clot passage
Urgency	Slight, no medication	Moderate, requiring local anesthetic (including bladder spasm)	Severe requiring local anesthetic	—
Incontinence	<weekly episodes	<daily episodes	Pads/undergarments/ day	Refractory

**Table 2:** Patient's characteristics for all patients and according to prescription group.

Characteristic		Prescription group		
	All (*n* = 126)	74R72 (*n* = 21)	76R74 (*n* = 19)	78R76 (*n* = 86)
Age (years)	66 (41–79)	62 (51–76)	65 (53–75)	66 (41–79)
Followup (months)	48 (24–132)	60 (24–132)	84 (24–108)	45 (24–96)
PSA level (ng/mL)	19 (4–302)	26 (8–150)	20 (4–90)	14 (4–302)
Gleason score				
2–6	49 (39)	9 (43)	11 (58)	29 (34)
7 (3 + 4)/(4 + 3)	37 (29)	7 (33)	5 (26)	25 (29)
8–10	39 (31)	5 (24)	3 (16)	31 (36)
Unknown	1 (1)	—	—	1 (1)
Tumor stage				
T1	17 (13)	2 (10)	4 (20)	11 (12)
T2	40 (32)	9 (43)	2 (11)	29 (34)
T3	60 (48)	7 (33)	12 (64)	40 (48)
T4	9 (7)	3 (14)	1 (5)	6 (6)
Node stage				
pN0	54 (43)	9 (43)	3 (16)	42 (49)
Androgen deprivation				
Yes	116 (92)	14 (67)	17 (89)	85 (99)
No	10 (8)	7 (33)	2 (11)	1 (1)

**Table 3:** Planning parameters for all patients and according to prescription. CTV: clinical target volume; Gy: Gray; PTV: planning target volume; R40 and R60: percentage of the rectal volume receiving a dose of 40 and 60 Gy, respectively; R_mean_: mean dose to the rectum, B_max_ and B_mean_: maximal and mean dose to the bladder.

	All (*n* = 126)	74R72 (*n* = 21)	76R74 (*n* = 19)	78R76 (*n* = 86)
CTV volume (cc)	61 (22–180)	86 (26–146)	68 (28–112)	53 (22–180)
Minimum CTV dose (Gy)	73 (55–77)	68 (55–70)	72 (67–74)	73 (68–77)
Median CTV dose (Gy)	78 (72–83)	76 (72–78)	77 (72–82)	79 (74–83)
PTV volume (cc)	155 (48–347)	250 (121–347)	226 (100–289)	123 (48–296)
Minimum PTV dose (Gy)	69 (52–73)	65 (52–68)	67 (65–70)	69 (64–73)
Median PTV dose (Gy)	77 (70–82)	74 (70–76)	75 (72–80)	78 (75–82)
R40	71 (30–97)	90 (88–90)	87 (57–97)	68 (30–94)
R60	43 (22–90)	64 (29–90)	54 (26–69)	40 (22–63)
R_mean_	51 (33–71)	57 (39–65)	54 (37–62)	49 (33–71)
B_max_	79 (72–82)	78 (72–79)	79 (76–82)	79 (76–82)
B_mean_	43 (13–72)	49 (19–68)	58 (16–65)	40 (13–72)

**Table 4:** Late gastrointestinal and genitourinary toxicity for all patients and according to prescription group.

Late GI toxicity				
	All (*n* = 126)	74R72 (*n* = 21)	76R74 (*n* = 19)	78R76 (*n* = 86)
Grade 1	52 (41)	9 (43)	8 (42)	35 (41)
Grade 2	20 (16)	6 (29)	2 (11)	12 (14)
Grade 3	2 (2)	0	1 (5)	1 (1)

Late GU toxicity				
	All (*n* = 126)	74R72 (*n* = 21)	76R74 (*n* = 19)	78R76 (*n* = 86)

Grade 1	55 (44)	11 (52)	7 (37)	37 (43)
Grade 2	26 (21)	1 (5)	4 (21)	21 (24)
Grade 3	7 (6)	3 (14)	1 (5)	3 (4)
